# The impact of informal control on the innovation performance of female technology professionals from the perspective of role pressure

**DOI:** 10.3389/fpsyg.2024.1378056

**Published:** 2024-10-29

**Authors:** Xiangfei Zeng, Mengyan Cao, Jingjing Hu, Wenpei Zhang

**Affiliations:** School of Business, Anhui University of Technology, Ma’anshan, China

**Keywords:** informal control, role pressure, innovation performance, environmental turbulence, female technology professionals, Chinese STEM field

## Abstract

**Background:**

With the rapid advancement of the technology industry, particularly in STEM fields, female professionals have increasingly become key drivers of innovation. Despite this, existing research has seldom examined the psychological impact of informal control on their innovation performance. Therefore, this study distributed questionnaires to female technology professionals in China’s STEM field to investigate the effect of informal control on their innovation performance from a psychological perspective. It further explored the mediating role of role pressure and the moderating role of environmental turbulence in this relationship.

**Methods:**

This study primarily utilized AMOS 24.0 to develop structural equation models, and employed PROCESS 24.0 and SPSS 26.0 for data analysis purposes.

**Results:**

The findings reveal that informal control positively predicted the innovation performance of female technology professionals in STEM field. Role pressure partially mediates this relationship, while environmental turbulence positively moderates the relationship between informal control and innovation performance among female technology professionals in STEM field.

**Discussion:**

Theoretically, this research enriches the individual-level approaches to enhancing management control effectiveness. Practically, it aids managers in focusing on the psychological well-being of female technology professionals in STEM field, thereby facilitating the judicious selection of management control methods. The study’s conclusions aim to provide logical guidance for enterprises to further strengthen their attention and support for female technology professionals in STEM field. Simultaneously, it offers a theoretical foundation for enhancing their innovative capabilities.

## 1 Introduction

The participation of female technological professionals in the labor market has been on a steady incline, positioning them as a pivotal contributor to socio-economic advancement. In particular, female professionals within STEM disciplines have risen to prominence as critical talent assets globally.^1^ Empirical evidence suggests that gender diversity within the STEM arena fosters enhanced team communication, superior financial outcomes, and augmented personal innovation performance among employees (Chen et al., 2021; Townsend and Busenitz, 2015). The women representation remains disproportionately low while the continuous enhancement of their power and status in STEM fields. At the “WLF She Forum “of the 6th World Laureates Forum held in 2023, Irina Bokova, former Director-General of UNESCO, highlighted that women continue to be underrepresented in scientific research and decision-making positions. Globally, women occupy only 33% of research positions and a mere 24% of leadership roles in scientific research. More alarmingly, women face even greater inequalities in the digital age, a situation that warrants particular attention (Falk and Hagsten, 2022). Nevertheless, studies have also pointed out that, deriving from gendered and social role discrepancies, women in STEM endure augmented occupational pressures and diminished work flexibility (Kossek et al., 2017). Female technological professionals face a multifaceted challenge as they navigate the concurrent demands of childcare, domestic education, and career responsibilities. This complex interplay of roles inevitably leads to a significant depletion of their psychological resources (Singh et al., 2018). A substantial number of STEM women, owing to career exigencies, forfeit, postpone, or reduce childbirth relative to their desires, significantly impacting their mental well-being (Paksi et al., 2022). Moreover, under identical circumstances, STEM women are often subjected to more rigorous scrutiny compared to their male counterparts. There exists a pervasive belief that women are unfit or less suited for STEM research tasks, which manifests as a recurrent threat to their professional identity. Many individuals experience an internal cognitive dissonance reconciling their gender and professional identities (Schmader, 2023).

Relative to their male counterparts, female technological professionals encounter additional challenges in the innovation process. Women display heightened sensitivity to the influence of senior management during innovation and necessitate augmented social support (Wang et al., 2013). The relationship between innovation and intrinsic variables (such as self-confidence, empowerment, and social processes) may be more pronounced for women (Nair, 2020). Under these circumstances, female technology professionals are more susceptible to role strain within their occupations, precipitating work-induced role overload and conflict, impressing their innovative performance (Bedford et al., 2022; Lin et al., 2022). Women possess qualities of rigor, meticulousness, and creativity, which are of paramount importance in scientific research (Denizci Guillet et al., 2019). However, it is an inescapable historical reality and an indisputable fact that women engaged in STEM fields must endure greater hardships and demonstrate enhanced resilience (Amon, 2017). Currently, the number of female scientific and technological personnel in China has reached 40 million, accounting for over 45% of the total workforce in this sector. Moreover, in China’s National Key Research and Development Program projects, there are more than 6,000 female project leaders. Consequently, the question of how to effectively enhance the innovative performance of female STEM professionals in China emerges as a critical issue demanding urgent resolution.^2^

Management control is the process by which organizational managers effectively acquire and utilize organizational resources to achieve organizational objectives (Simons, 2019). It not only serves as a crucial antecedent variable for innovative performance but also acts as a buffer zone between social institutions and individual actions (Hopwood, 1973). Scholars have not reached a consensus in their research on the relationship between management control and innovative performance, with three main perspectives emerging: inhibitory, promotional, and inverted U-shaped. The inhibitory view posits that management control is detrimental to the development of new products in enterprises, as formal control emphasizes rules and efficiency, potentially reducing employee innovation performance (Tang et al., 2020). Process and outcome controls focus on tight budgetary targets and meticulous output monitoring, lacking the flexible structures and processes necessary to address uncertain environments. This inability to effectively implement dynamic strategic change objectives leads to myopic innovation decision-making (Sandalgaard and Nielsen, 2018). Formal control restricts managers’ access to work resources (Lidia, 2014), exacerbating the conflict between achieving control objectives and organizational innovation, increasing work pressure (Bedford et al., 2022), and affecting organizational innovation decisions (Lian and Wang, 2015). The promotional view argues that when management control is used as an empowerment system or interactive system, it helps create dynamic tension relationships, thereby supporting employee innovation processes (Revellino and Mouritsen, 2023; Müller-Stewens et al., 2020). Formal control can leverage experience and technical resources to make process changes, thus developing dynamic capabilities (Dentoni et al., 2016). This enables organizations to execute predetermined performance goals more accurately and efficiently, leading the trend of innovative change (Townsend and Busenitz, 2015). The inverted U-shaped view suggests that the impact of management control on innovative performance has a threshold. Managers can enhance corporate innovation by setting performance goals, but excessive performance pressure in management control can inhibit R&D activities (Zhou et al., 2019). Both overly lenient and overly strict process controls adopted by managers negatively affect employee innovation (Zhou et al., 2019; Rijsdijk and van den Ende, 2011).

We observe two key points: First, most literature focuses solely on the impact of formal control within management control on innovation, neglecting the role of informal control. Unlike the West, China’s unique socio-cultural background inevitably profoundly influences management control design. Management control has not only a technical dimension but also a managerial context dimension, which is more subject to the influence of informal control (Osma et al., 2022). Informal control in management control focuses more on factors involving social psychology and behavioral science behind specific management functions, especially informal management control factors such as ethics, cultural norms, and communication mechanisms in organizations. Informal control emphasizes trust, communication, cooperation, and sharing, all of which are closely related to female characteristics (Huang and Aaltio, 2014). Second, existing research mostly explores the impact of management control on innovation at the organizational level, rarely focusing on the role of informal control at the individual psychological level (stress, emotions, etc.). The individual psychological contingency theory posits that by examining the relationship between management control, individual psychological states and behaviors, which can elucidate the operation and effects of management control at the personal level (Luft and Shields, 2010). In cognitive psychology, stress is often used to explain and predict the effects of management control (Birnberg et al., 2006). According to the Job Demands-Resources (JD-R) model, role stress, as an important aspect of stress, represents a hindering organizational demand (Bakker and Demerouti, 2014; Crawford et al., 2010), making it difficult for employees to have sufficient resources to engage in innovative activities (Bakker and Demerouti, 2014). Informal control helps female scientific and technological workers better communicate with organizations and team members (Grabner et al., 2018), and assists them in recognizing their obligations and responsibilities in various work roles (Segarra-Ciprés et al., 2019), aligning with the psychological quality requirements of innovative activities for female employees (Leidner et al., 2020).

Additionally, contingency theory suggests that the innovative impact of management control is susceptible to interference from contextual factors (Galbraith, 1973). Studies have shown that higher levels of environmental turbulence can enhance individual innovation awareness (Bodlaj and Čater, 2019) and also amplify the facilitative role of systemic information on product innovation (Henri and Wouters, 2020). Therefore, it is imperative to investigate the relationship between informal control and the innovative performance of women in the STEM field from the perspective of role pressure, as well as the contextual factors that influence this relationship.

Building on this, this paper introduces the Job Demands-Resources (JD-R) model as a foundational framework, aiming to explore the pathways through which informal control can enhance the innovative performance of female scientists and technicians in China’s STEM field from the perspective of role pressure, and to propose policy recommendations. The potential contributions of this paper are as follows: Theoretically, it facilitates the clarification of the theoretical threads under the research topic of women in the STEM field, expands the literature on the impact of management control on individual psychological states, complements research on the influencing factors of role stress, and enriches the understanding of personal-level approaches to enhancing the effectiveness of management control. Practically, it promotes diversification in corporate management control methods, aids in prompting managers to pay attention to the psychological well-being of female scientific and technical personnel, and guides the rational selection of management control strategies. The findings are intended to provide logical guidance for enterprises to further strengthen their focus and support for female scientists and technicians, while also offering a theoretical basis for enhancing their innovative capabilities. This translation is crafted to be smooth, logically clear, and in alignment with the standards of a master’s thesis.

The structure of this paper is as follows: the second section conducts theoretical analysis and proposes hypotheses, the third section introduces the research methods, the fourth section presents data analysis and results, and the fifth section discusses the findings and outlines the limitations.

## 2 Theoretical analysis and research hypothesis

### 2.1 The effect of informal control on the innovation performance of female technological professionals

The Job Demands-Resources (JD-R) model is a predominant theoretical framework for studying the impact of work characteristics on occupational psychological health (Lesener et al., 2019). This model is widely utilized in research on workplace stress and exhaustion, illuminating the complex interplay between occupational requirements and available resources, as well as their subsequent effects on employee output (Ali et al., 2019; Kwon and Kim, 2020). The JD-R theory posits that an organization’s drive for innovation can impact individual work drive, potentially leading to increased work stress (Kwon and Kim, 2020). This model categorizes the factors influencing workers’ physical and mental health and work conditions into two main types: job demands and job resources. It can be used to explain and predict work-related behaviors such as work engagement.

As per the JD-R model, job demands imply physical and psychological efforts exerted by individuals (Scholze and Hecker, 2024) and are associated with physical and mental exhaustion, such as role ambiguity and role conflict (Van Slooten et al., 2024). When individuals use their personal resources to cope with job demands (Morin et al., 2023), it may lead to resource imbalance, affecting work engagement and job performance (Kloutsiniotis and Mihail, 2020). On the other hand, job resources refer to the resource individuals obtain through information feedback, autonomy, etc., which help alleviate the consumption associated with job demands, motivate personal learning and development, and assist in achieving personal goals (Bullini Orlandi et al., 2024).

Formal control exacerbates the adverse impact of pressure on female technology professionals. It entails a hierarchical management approach characterized by a “top-down” structure with limited transparency. The absence of horizontal communication among departments poses a challenge for aligning individual innovative endeavors with the actual resource allocations within each department. Significantly, during periods of uncertainty, the restrictive control measures implemented through formal control further intensify the perceived role pressure experienced by female technology professionals. In such circumstances, female professionals in the technology sector expend additional work resources, resulting in prolonged pressure and adverse outcomes such as resignations. Replenishing these work resources proves challenging under formal control, leading to a resource deficit for female technology professionals involved in innovative endeavors. This scarcity hampers their initiative and creativity (Chen et al., 2018; Lyngsie and Foss, 2017), impacting innovation performance. Within the traditional paradigm that underscores control and conquest, the ongoing replication of male discourse dominance marginalizes females, making them comparatively reticent and less likely to engage in integrated discourse. In this context, managers striving to advance an innovative paradigm may fall short of achieving the anticipated outcomes (Perra et al., 2017).

Within the context of informal control, managers who engage in extensive and thorough communication within the corporate can provide female technology professionals in STEM field with the necessary information resources, which can facilitate individual innovation (Van Oosten et al., 2017). Managers can offer female technology professionals a platform for open, face-to-face communication. This approach enables female technology professionals to access more specific and detailed operational information directly from the enterprise front lines, allowing the enterprise resources to better align with the individual’s innovative requirements. Moreover, this cooperative atmosphere under informal control, characterized by shared information, is conducive to the processing and absorption of information by female technology professionals in STEM field, thereby increasing the utilization of information resources and fostering the individual’s innovative performance.

Moreover, informal control satisfies female technology professionals in STEM field cognitive needs and mindfulness levels. According to Cacioppo and Petty (1982), an individual’s need for cognition is manifested by their desire for involvement and learning. On the one hand, regular and comprehensive internal communication and learning about uncertainties within enterprise promote knowledge sharing and collective growth among organizational members, thereby enhancing the creativity of female technology professionals in STEM field (Ashiru et al., 2022) and maximizing the potential for individual innovation. On the other hand, it fosters female technology professionals in STEM field voice behavior, positively impacting individual innovative performance. Attribution theory suggests that an individual’s initial perceptions of events can influence their cognition and behavior (Martinko and Mackey, 2019). Informal control provides female technology professionals in STEM field with the necessary informational and cognitive resources, enhancing their capacity to perform their roles effectively. In the face of unforeseen events, female technology professionals in STEM field are more likely to perceive innovation tasks as challenge demands rather than hindrance demands (Sahi et al., 2023). Based on the above analysis, this article proposes the following hypothesis.

*H1*: Informal control positively predicts innovation performance of female technology professionals.

### 2.2 The impact of informal control on the role pressure of female technological professionals

Role pressure emerges when individuals struggle to comprehend or fulfil the expectations associated with their roles, creating a negative perception within the context of work-related pressure. It encompasses two dimensions: role ambiguity and role conflict (Rizzo et al., 1970). At present, the research on the influence factors of role pressure mainly focuses on two aspects: individual and organization. At the individual level, gender and position (Carillo et al., 2021; Hall, 1989), tenure, and age (Hessels et al., 2017; Hayes et al., 2021) impact an individual’s encounter with role pressure.

On the organizational level, factors like the mode of authorization control (Bedford et al., 2022), leadership style, and capabilities (Li et al., 2020) also exert influence on the role pressure experienced by female technology professionals.

It is difficult for female technology professionals in STEM field to obtain adequate resources in a formal, controlled atmosphere, which will exacerbate the perception of role pressure. In this mode, the upper and lower levels lack adequate communication, and the participation of members is low. As a result, the innovation work cannot reflect the consensus of all parties and is contrary to the values of superior leaders. Women prefer to be appreciated for a job well done. From a work perspective, the innovative abilities of female employees are easily suppressed by an unfavorable organizational climate. Informal control enhances their identification with and commitment to the corporate, reducing the likelihood of negative behaviors such as psychological withdrawal and turnover in the workplace. Organizational support can provide psychological assurance to a certain degree; when female employees feel supported and cared for by the corporate, particularly when needing assistance, their job satisfaction tends to increase, and pressure levels correspondingly decrease (Wu et al., 2021).

On the one hand, informal control mechanisms enable managers to mitigate role conflicts among female technology professionals in STEM field. When managers offer extensive support and guidance to these professionals, they foster increased confidence and optimism, diminish feelings of role conflict and overload, and ultimately enhance psychological safety. Both the process of employee innovation and the realization of innovation performance have certain risks. For self-protection, female technology professionals are willing to take risks to innovate their working methods and improve their innovation performance only in an environment full of support and security. Effective communication reinforces innovation intentions and behaviors among female technology professionals. In instances of role conflicts experienced by female technology professionals, enterprise through guiding culture and values shapes employees’ psychological perceptions, leading to heightened feelings of responsibility and identification. The perceived fairness in performance evaluations enhances innovative behavior among female technology professionals (Newman et al., 2018). Engaging in diverse forms of internal communication within the organization allows female technology professionals to share high-quality information resources, facilitating a rational allocation of resources aligned with innovation goals and mitigating role conflicts. A reduced power distance, coupled with regular and thorough communication addressing key issues among employees, promotes continuous learning of new knowledge and skills, transforms implicit knowledge into explicit forms, and strengthens their ability to attain innovation goals (Nguyen et al., 2022).

On the other hand, through informal control, managers can mitigate role ambiguity among female technology professionals. Information exchange within the framework of informal control is characterized by greater transparency and a higher frequency of feedback. This not only assists female technology professionals in strategically planning their tasks, providing clarity on work objectives with a long-term perspective, but also facilitates the swift identification and resolution of issues within innovative work, specifies individual behaviors, and consequently reduces role ambiguity (Lian and Wang, 2015). Concurrently, female technology professionals are empowered to concentrate on the entire innovation process, comprehend the consequences of innovative actions, adapt flexibly to uncertain environments, and diminish role ambiguity (Riedl and Thomas, 2019). By clearly delineating responsibilities aligned with role expectations and acquiring the necessary resources to fulfil these expectations, female technology professionals alleviate role ambiguity. Managers, employing effective communication strategies, can exhibit empathy and tolerance, displaying a relatively more accommodating attitude towards the setbacks experienced by female professionals, thus can alleviate hesitation in their innovative decision-making processes and contribute to making well-founded, clever decisions (Kroll, 2019). Based on the above analysis, the following hypothesis is proposed in this paper.

*H2*: Informal control negatively predicts the role pressure level of female technology professionals.

### 2.3 The mediating role of role pressure

Role pressure, acknowledged as an inhibitory pressure (Cavanaugh et al., 2000), is linked to various health conditions (O'Connor et al., 2021), primarily impacting job efficiency and negatively influencing individual innovation performance (Su et al., 2022). Female technology professionals require substantial personal resources to navigate role pressure successfully. However, within a formal control framework, acquiring adequate information and cognitive resources proves challenging (Wohlgemuth et al., 2019), affecting their involvement in innovative activities. Concurrently, the emphasis by higher-level leaders on the personal innovation goals of female technology professionals exacerbates the adverse effects of role pressure, presenting a formidable challenge to enhancing individual innovation performance. Role pressure can instigate self-doubt among female technology professionals, confusing them and impeding their ability to allocate sufficient time and energy to innovative work (Wang R. et al., 2023; Wang X. et al., 2023). Therefore, informal control could enhance individual innovation performance by influencing the role pressure of female technology professionals. When all departments can fully engage in dialogue, learning, collaborating and sharing information, female technology professionals, thus relieving their role pressure and lowering their sense of need for work resources, so that they will have more adequate resources to be used in the innovation activities of the enterprise. At the same time, when the information and cognitive resources of female technology professionals are satisfied, they will also participate in the innovation work more actively. Drawing on the above analysis, this paper posits the following hypotheses.

*H3*: Role pressure mediates the relationship between informal control and the innovation performance of female technology professionals.

### 2.4 The regulatory role of environmental turbulence

Environmental turbulence refers to the frequency of unpredictable and highly diverse events within an organizational setting, characterized by a strong degree of uncertainty (Tsai and Yang, 2014). Following contingency theory, the impact of management control on employee innovation performance can be disrupted by situational factors (Lewis et al., 2023). Environmental turbulence compels continuous interaction between internal and external corporate members to adapt to environmental changes, thereby increasing the information demand (Wang R. et al., 2023; Wang X. et al., 2023). Informal control can precisely fulfil this resource necessity, aligning with the dynamic external environment. Informal control facilitates dialogue among managers and female STEM employees, as well as between the female STEM employees themselves, fostering information exchange and enabling them to address the constantly evolving environment flexibly, thus reducing innovation inefficiencies due to the neglect of dynamic surroundings (Müller-Stewens et al., 2020). It can be postulated that in more instable environments, managers are likely to employ informal control more actively and frequently, thereby enhancing the positive impact of informal control on the innovation performance of female STEM employees and easing their role pressure. Furthermore, in line with the views of certain scholars (Hayes and Coutts, 2020), the mediating effect can also be moderated when either the first or second half of the mediating pathway is modulated by a moderator variable. Integrating the above analysis and the previously mentioned Hypotheses 1 and 2, this study posits the following hypotheses.

*H4*: Environmental turbulence moderates the relationship between informal control and innovation performance of female technology professionals.

*H5*: Environmental turbulence moderates the relationship between informal control and role pressure.

*H6*: When the degree of environmental turbulence is high, informal control has a more substantial effect on the innovation performance of female technology professionals by influencing role pressure.

**Figure 1:**
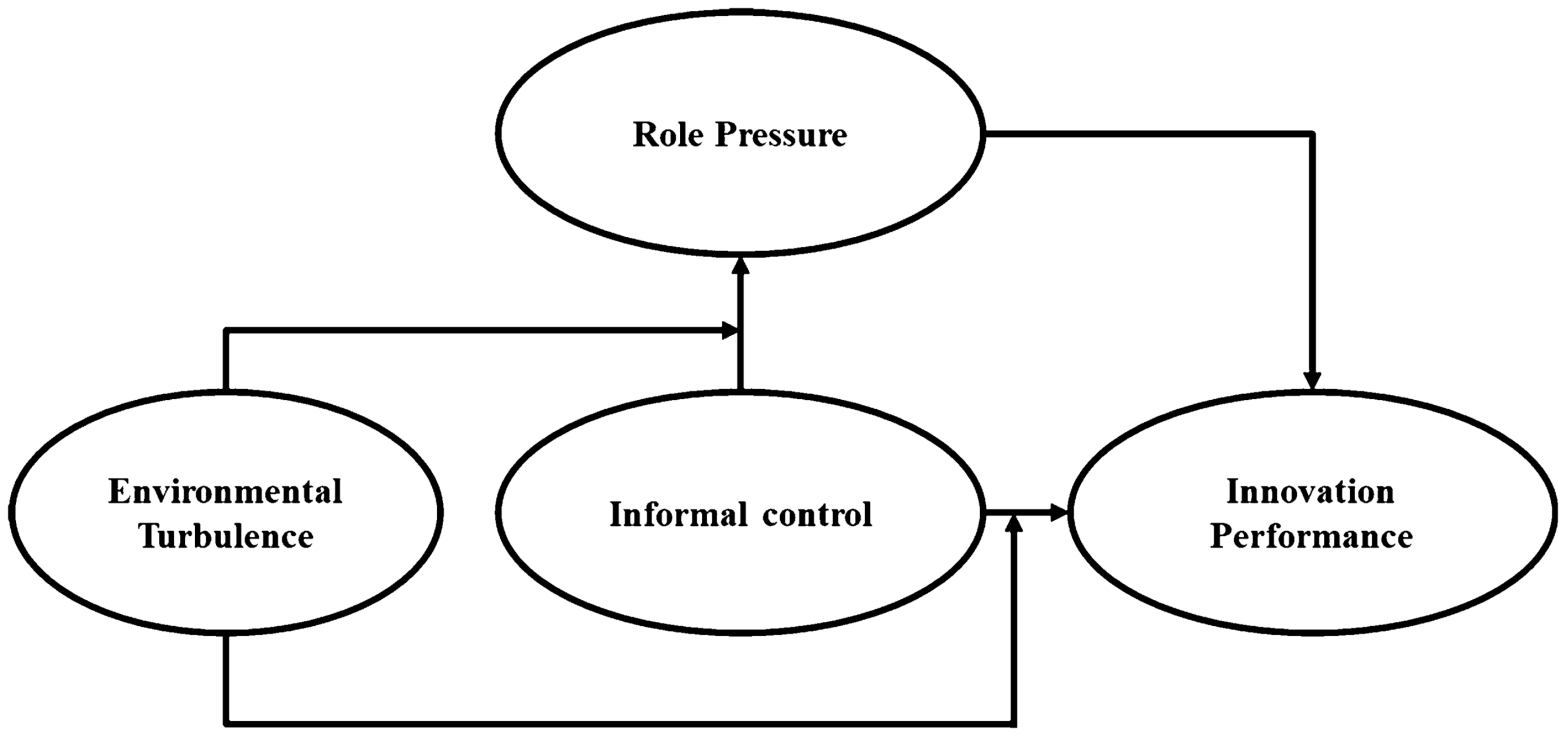
Frame model diagram.

The research model of this paper is shown in Figure 1.

## 3 Research methods

### 3.1 Sample selection and data collection

Based on a comprehensive review of existing literature, we formulated a complete research design framework and drafted an initial questionnaire. To ensure the reliability and validity of Western-derived concepts within the Chinese context, we conducted targeted visits to three enterprises in a Chinese city, engaging in interviews with female technology professionals in STEM field. The interviews primarily focused on aspects such as enterprise management control methods, personal performance objectives, work environment, and role-related pressures. During these interviews, Engineer A remarked, “The enterprise’s formal control measures are sometimes excessively rigid, lacking any flexibility. On one occasion, when dealing with an urgent document, we were still required to adhere to the prescribed procedures, which felt not only time-consuming but also gave a sense of distrust, as if under constant surveillance. With my child being only 3 years old, I distinctly feel overwhelmed at work.” R&D Manager B shared, “My superior frequently organizes team brainstorming sessions and discussions, fostering a vibrant atmosphere. Her communication with us is exceptionally frequent, promptly addressing both work-related and personal issues, making the enterprise feel more akin to a family. Particularly during the extraordinary period of the COVID-19 pandemic, she was able to coordinate our flexible work arrangements remotely.” Programmer C expressed, “In this field, the pressure on female programmers is considerable. I need to strive for technical excellence while simultaneously confronting gender biases. I’m often asked questions like ‘How did you manage that?’ as if women are inherently incompatible with programming. Some even suggest that I’m more suited for non-development roles such as documentation.” These research findings indicate that Chinese enterprises still have substantial room for improvement and a pressing need for enhancement in balancing formal and informal controls, gender equality awareness, and the development and support of female STEM talent. Building on these insights, we invited five experts in the field of management control research to discuss the questionnaire and meticulously revise the wording of the items, ultimately finalizing the questionnaire.

The primary target respondents for this study were female technology professionals in China’s STEM field. We executed a large-scale survey in November 2023. For online distribution, all questionnaires were disseminated through the reliable online platform Credamo (www.credamo.com).^3^ In order to ensure that eligible respondents fill in the questionnaire, we have limited the respondents’ conditions in the questionnaire. A total of 300 online questionnaires were distributed, accompanied by a confidentiality assurance statement to foster open responses. Additionally, to maintain data validity and prevent duplicate submissions, IP addresses were restricted to one survey completion each. All reactions were anonymized. Of the 300 collected surveys, after excluding incomplete, excessively brief/lengthy, or consistently uniform responses, a final dataset of 255 valid responses was retained, yielding an effective response rate of 85%. Refer to the Appendix for questionnaire items, and Table 1 presents the sample’s demographic characteristics. Data analysis utilized SPSS 26.0, Amos 24.0, and Process 24.0.

**Table 1:** Sample statistics.

Characteristic	Survey number	Percent
Ownership		
State enterprise	51	20.0
private enterprise	82	32.2
corporate corporation	81	31.7
foreign company	41	16.1
Number of employees		
21–300	70	27.5
301–1,000	148	58.0
>1,000	37	14.5
Age (year)		
21–25	1	0.4
26–30	63	24.7
31–35	127	49.8
36–40	44	17.3
41–45	9	3.5
>46	11	4.3
Industry type		
High-tech	204	80.8
Non-high-tech	51	20.0
Children		
No children	86	33.7
Have children	169	66.3

### 3.2 Variable measurement

The predominant approach in this study involved using established and widely recognized foreign scales to measure five latent variables. A translation-back-translation process was implemented to ensure the validity of these scales. The questionnaire employed a 7-point Likert scale, where one indicated “strongly disagree” and seven indicated “strongly agree.”

#### 3.2.1 Informal control (IC)

Drawing upon the work of Jaworski et al. (1993), we incorporated two dimensions: social control and cultural control, comprising a total of ten items. The social control dimension includes five items, exemplified by statements such as “Managers encourage employees to cooperate with each other “The cultural control dimension also consists of five items, with examples like “The employees of the enterprise have a high degree of participation in the work “The Cronbach’s *α* coefficient for this scale is 0.899.

#### 3.2.2 Innovation performance (IP)

This study adopted the innovation performance scale developed by Moqbel and Nah (2017), which encompasses five items. These items cover various aspects of innovation, including the generation of innovative ideas, exploration of new work methods or technologies, practical application of innovative ideas, recognition of innovative ideas by management, and innovative solutions for problem-solving. An example item is “Create new ideas for improvements.” The Cronbach’s α coefficient for this scale is 0.830, indicating good reliability.

#### 3.2.3 Role pressure (RP)

Based on the research findings of Rizzo et al. (1970) and Bedford et al. (2022), we incorporated two dimensions of role pressure: role conflict and role ambiguity, totaling nine items. The measurement of role conflict utilizes four items, addressing aspects such as work demands, contradictions in task assignments, and whether one receives praise or criticism at work. An example item is “I have to work on things that should be done differently.” The measurement of role ambiguity employs five items, covering areas such as ambiguity in job scope, work responsibilities, work objectives, and time allocation. An example item is “I do not have clear, planned goals.” The Cronbach’s α coefficient for this scale is 0.909.

#### 3.2.4 Environmental turbulence (ET)

Leveraging the scale devised by Miller and Friesen (1982), the current study evaluates environmental turbulence through five items associated with the corporate marketing strategy: the rate of updating products or services, the pace of technological advancements, actions taken by competitors, and customer demand preferences. The Cronbach’s alpha coefficient for this scale is 0.952.

#### 3.2.5 Control variable

Taking into account the features of China’s economic transformation, the innovation performance of corporate is affected by the nature of their ownership (Ren et al., 2022). Furthermore, Chandy and Tellis (2000) research indicates that the innovation capability of corporate differs based on their size. Consequently, we designate the nature of corporate ownership (OS) and the number of employees (NE) as control variables.

## 4 Result

### 4.1 Common method bias

Univariate testing of the Harman was conducted using SPSS 26.0. The results revealed that the cumulative variance contribution reached 64.552%, with the initial factor variance representing 30.558% of the total variance, falling below the 40% threshold. This outcome implies a low likelihood of common method bias in the study. Examination of Table 2 indicates that the four-factor model (χ2/df = 2.219, RMSEA = 0.069, SRMR = 0.059, CFI = 0.911, TLI = 0.902) exhibited a significantly better fit than the one-factor model (χ2/df = 7.015, RMSEA = 0.154, SRMR = 0.147, CFI = 0.548, TLI = 0.515). These statistical tests collectively suggest the absence of substantial methodological bias in our study.

**Table 2:** Results of confirmatory factor analysis.

	χ^2^/df	RMSEA	SRMR	CFI	TLI
Four-factor model IC, RP, IP, ET	2.219	0.069	0.059	0.911	0.902
Three-factor model IC + RP, IP, ET	3.200	0.093	0.069	0.836	0.823
Two-factor model IC + RP + IP, ET	3.600	0.101	0.088	0.805	0.790
Single factor model IC + RP + IP + ET	7.015	0.154	0.147	0.548	0.515

### 4.2 Reliability and validity test

As previously indicated, the Cronbach’s alpha coefficients for the scales range from 0.830 to 0.952, signifying that the scales exhibit satisfactory reliability. Regarding convergent validity, the results from the confirmatory factor analysis in Table 2 reveal a well-fitting model with χ2/df = 2.219, RMSE = 0.069, SRMR = 0.059, CFI = 0.911, and TLI = 0.902. Additionally, Table 3 illustrates that the Average Variance Explained (AVE) values for each latent variable range from 0.58 to 0.67, all surpassing the 0.5 threshold. Collectively, these findings indicate convergent solid validity for the scale. Furthermore, as evidenced in Tables 2, 3, the fit indices for the four-factor model (χ2/df = 2.219, RMSEA = 0.069, SRMR = 0.059, CFI = 0.911, TLI = 0.902) outperform those of alternative models. The square roots of the AVE for each latent variable also exceed the correlation coefficients between the variable and others, suggesting robust discriminant validity among the four constructs in this study. Lastly, reiterating earlier, the scales utilized in this study have proven maturity and widespread usage. With the guidance of relevant experts and adjustments aligned with the research background, objectives, and context, the scales exhibit strong content validity.

**Table 3:** Mean value, standard deviation and correlation analysis of research variables.

	Mean	SD	AVE	1	2	3	4	5	6
OS	2.60	1.13							
NE	2.13	0.63		−0.03					
IC	5.97	0.59	0.58	−0.05	0.21***	**0.76**			
RP	2.08	0.89	0.58	−0.05	−0.15*	−0.71***	**0.76**		
IP	5.57	0.68	0.67	−0.13*	0.46***	0.68***	−0.64***	**0.82**	
ET	5.22	1.47	0.54	−0.02	0.34***	0.17***	−0.26***	0.52***	**0.73**

*p < 0.05, **p < 0.01, ***p < 0.001; the bold words in the table represent the square root of each variable AVE.

### 4.3 Descriptive statistical analysis

Table 3 displays each variable’s mean values, standard deviations, and correlation coefficients. The table reveals a noteworthy negative correlation between the utilization of informal control by corporate and employee role pressure (*r* = −0.71, *p* < 0.001), coupled with a significant positive correlation with innovation performance (*r* = 0.68, *p* < 0.001). Furthermore, employee role pressure significantly correlates negatively with innovation performance (*r* = −0.64, *p* < 0.001). These findings closely correspond to the anticipated hypotheses and substantiate subsequent analyses.

### 4.4 Intermediate model checking

First, the PROCESS program Model4 (simple mediation model) of Hayes and Coutts (2020) was used to test the mediation effect of employee role pressure in the relationship between informal control and innovation performance under the condition of controlling the equity nature and corporate size. The results, presented in Table 4, demonstrate that informal control significantly predicts innovation performance (*B* = 0.71, SE = 0.05, *t* = 14.83, *p* < 0.001) and also considerably predicts employee role pressure (*B* = −1.07, SE = 0.07, *t* = −15.87, *p* < 0.001). Thus, our proposed Hypotheses H1 and H2 find empirical support. Furthermore, employee role pressure emerges as a significant predictor of innovation performance (*B* = −0.25, SE = 0.04, *t* = −5.93, *p* < 0.001). Even with the introduction of the mediating variable role pressure, the predictive impact of informal control on innovation performance remains substantial (*B* = 0.44, SE = 0.06, *t* = 6.97, *p* < 0.001).

**Table 4:** Mediating model test of role pressure.

Regression equation (*N* = 255)		Fitting the index			Coefficient significance		
Outcome variable	Predictive variable	*R*	*R* ^2^	*F*	*B*	SE	*t*
IP		0.77	0.59	119.49			
	OS				−0.06	0.02	−2.40*
	NE				0.36	0.04	8.03***
	IC				0.71	0.05	14.83***
RP		0.72	0.51	88.16			
	OS				−0.06	0.03	−1.85
	NE				−0.01	0.06	−0.05
	IC				−1.07	0.07	−15.87***
IP		0.79	0.64	110.58			
	OS				−0.07	0.02	−3.22**
	NE				0.36	0.04	8.54***
	IC				0.44	0.06	6.97***
	RP				−0.25	0.04	−5.93***

Moreover, the direct impact of informal control on corporate innovation performance and the mediating influence of female employees’ role pressure, as indicated by bootstrap 95% confidence intervals for the upper and lower bounds (refer to Table 5), excluding 0. This implies that informal control not only directly predicts innovation performance but also anticipates it through the mediating mechanism of employee role pressure. Therefore, Hypothesis H3 receives support.

**Table 5:** Total effects, direct effects and intermediate effects.

	ES	SE	Lower	Upper	RES
Total	0.71	0.05	0.60	0.80	
Direct	0.44	0.11	0.21	0.65	61.97%
Indirect	0.27	0.11	0.06	0.49	38.03%

### 4.5 Regulating effect

**Figure 2:**
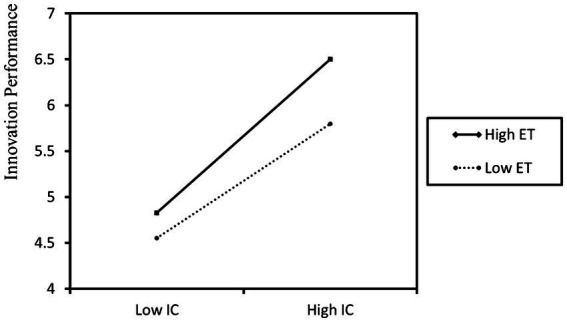
The moderating role of environmental turbulence in the relationship between informal control and innovation performance.

**Figure 3:**
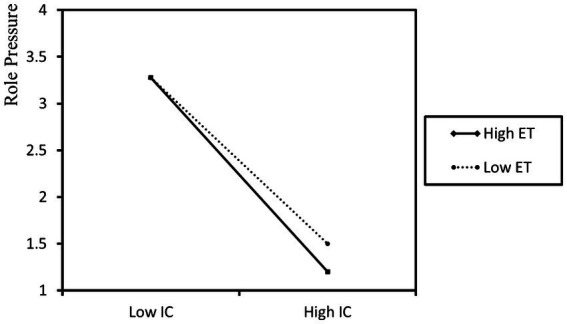
The moderating role of environmental turbulence in the relationship between informal control and role pressure.

The results of additional simple slope analysis, depicted in Figure 2, reveal that in situations of high environmental turbulence, the positive influence of informal control on innovation performance becomes more prominent. Figure 3 illustrates that when facing increased environmental turbulence, the detrimental effect of informal control on role pressure becomes more noteworthy. Hence, Hypotheses H4 and H5 find support (Table 6).

**Table 6:** Moderated mediation model tests.

Regression equation (*N* = 255)		Fitting the index			Coefficient significance		
Outcome variable	Predictive variable	*R*	*R* ^2^	*F*	*B*	SE	*t*
RP		0.74	0.55	61.70			
	OS				−0.07	0.03	−2.16*
	NE				0.07	0.06	1.08
	IC				−1.21	0.08	−14.39***
	ET				−0.11	0.03	−3.87***
	IC × ET				−0.10	0.03	−3.10**
IP		0.85	0.72	107.62			
	OS				−0.06	0.02	−3.14**
	NE				0.25	0.04	6.43***
	IC				0.57	0.07	8.23***
	ET				0.15	0.02	8.58***
	IC × ET				0.04	0.02	2.25*

*p < 0.05, **p < 0.01, ***p < 0.001.

The results of examining the moderating effect of environmental turbulence on the mediating role of employee role pressure are outlined in Table 7. The findings suggest that as the degree of environmental turbulence steadily increases, the mediating impact of role pressure in the positive relationship between informal control and employee innovation performance progressively strengthens (coefficients ranging from 0.17 to 0.22). Therefore, Hypothesis H6 is supported.

**Table 7:** Direct and intermediate effects at different levels of environmental turbulence.

	ET	ES	SE	Lower	Upper
	−1.48	0.50	0.06	0.39	0.61
Direct	0.00	0.57	0.07	0.43	0.70
	1.48	0.63	0.08	0.46	0.81
	−1.48	0.17	0.09	0.01	0.37
Indirect	0.00	0.20	0.10	0.01	0.40
	1.48	0.22	0.11	0.01	0.44

### 4.6 Further analysis

Subsequently, this study further examines the impact of informal control on the innovation performance of female technology professionals in STEM field based on two aspects: the type of enterprise (high-tech versus non-high-tech)^4^ and whether the female technology professionals in STEM field have children (Table 8).

**Table 8:** Heterogeneity test.

	Grouping and regression results								Group significance test		
	Unstd	SE	*P*	Std	Unstd	SE	*P*	Std	DF	CMIN	*P*
	High-tech				Non-high-tech						
IC- > IP	0.801	0.046	0.000***	0.726	0.351	0.136	0.013*	0.263	1.000	3.977	0.046*
IC- > RP	−1.052	0.071	0.000***	−0.728	−1.256	0.235	0.000***	−0.612	1.000	0.122	0.727
	No children				Have children						
IC- > IP	0.336	0.165	0. 045*	0.217	0.827	0.048	0.000***	0.801	1.000	0.115	0.735
IC- > RP	0.114	0.175	0.517	0.071	−1.220	0.063	0.000***	−0.833	1.000	4.233	0.040*

*p < 0.05, **p < 0.01, ***p < 0.001. Unstd, unstandardized coefficients; Std, standardization coefficient; DF, degree of freedom; CMIN, chi-square.

The findings reveal a statistically significant distinction (*p* < 0.05) in the influence of informal control on the innovation performance of female technology professionals in STEM field across high-tech and non-high-tech enterprise. Specifically, informal control exerts a more pronounced facilitating influence on the innovation performance of female technology professionals in high-tech enterprise (*p* < 0.001) compared to their counterparts in non-high-tech enterprise (*p* < 0.05).

A discernible variation (*p* < 0.05) emerges in the impact of informal control on the role pressure levels experienced by female technology professionals, depending on their parental status. Specifically, informal control significantly mitigates role pressure among female technology professionals with children (*p* < 0.001), while its suppressive effect on role pressure lacks statistical significance for those without children.

According to knowledge management theory, compared with non-high-tech enterprises, the core competitive advantage of high-tech enterprises resides in their capacity for innovation and the rapid transformation of knowledge (Huang et al., 2023). Within this context, informal control mechanisms facilitate knowledge sharing and collaboration, which are critical for fostering innovation. Female technology professionals may be more inclined to promote innovative thinking through cooperation and communication, and therefore, in such an enterprise culture and work atmosphere, their innovation performance is more effectively improved. Therefore, compared with non-high-tech enterprises, informal control plays a stronger role in promoting the innovation performance of female technology professionals in high-tech enterprises.

For the empirical results of whether female technology professionals have children, we make the following explanation according to the role theory. There exists a well-defined expectation regarding the maternal role for women with children, resulting in specific role pressures within both work and family contexts (Mensah, 2021). Informal control, such as flexible work arrangements and an emphasis on employee well-being, can assist mothers in better reconciling their professional responsibilities with motherhood, thereby alleviating role-related stress to some extent. In contrast, for women without children, societal expectations regarding their roles are less rigid and explicit. Consequently, the role pressures they encounter in the workplace are primarily driven by the demands of their positions and expectations related to career advancement. As such, informal control measures implemented by organizations may not adequately address these distinct needs, resulting in a negligible impact on their experiences.

## 5 Conclusion and discussion

### 5.1 Conclusion

This study investigates the influence of informal control on the innovation performance of 255 female technology professionals in the Chinese STEM field. The research explores the mediating role of role pressure and the moderating impact of environmental turbulence on this relationship. The key findings are as follows: (1) Informal control significantly and positively affects innovation performance while exerting a substantial negative influence on employee role pressure. The adverse impact of employee role pressure on innovation performance is also considerable. Even with the introduction of the mediating variable role pressure, the positive effect of informal control on innovation performance remains statistically significant; (2) In conditions of high environmental turbulence, the positive impact of informal control on innovation performance is amplified. Conversely, under elevated environmental turbulence, the negative effect of informal control on innovation performance becomes more pronounced.

### 5.2 Theoretical contribution

Firstly, by examining the relationship between management control and individual psychological states and behaviors, this study elucidates the operational mechanisms and effects of management control at the individual level, thereby enriching the personal-level approaches to enhancing management control effectiveness. The impact of specific types of management control practices on individual behavior depends not only on objective evaluations of their influence but also on how these practices are understood by individuals (Birnberg et al., 2006). This study advances management control studies from considering environmental changes as an external objective factor to an internal subjective psychological factor, shifting the analysis from the organizational level to the individual perception level.

Secondly, this study expands the literature on factors influencing current innovation performance. Although previous research has indicated that formal control affects enterprise innovation, no consensus has been reached (Jin and Wang, 2024; Müller-Stewens et al., 2020; Rijsdijk and van den Ende, 2011). Most literature focuses on the impact of formal management control on innovation, neglecting the role of informal control. Due to cultural backgrounds and national conditions, scholars lack systematic and comprehensive research on the pathways influencing individual innovation performance of female STEM professionals (Poggesi et al., 2020). This study analyzes effective pathways to enhance innovation performance for female STEM professionals from the perspective of role pressure, providing a better understanding and explanation of the operational effects of management control.

Thirdly, this study enriches research on role stress. In the field of management control research, individual perceptions of informal control are rarely addressed (Bedford et al., 2022). The results of this study demonstrate a negative correlation between informal control and role pressure among female STEM professionals, supplementing the antecedent variables affecting role stress. This also implies that informal control can address the resource depletion caused by job demands on individuals (Bakker and Demerouti, 2014), providing a theoretical basis for resolving psychological issues faced by female STEM professionals.

### 5.3 Management enlightenment

Firstly, enterprises need to recognize the importance of informal control in enhancing the innovation performance of female STEM professionals. During innovation activities, companies should strengthen informal control measures, enabling more women to better engage in scientific research and creating a fairer research atmosphere for them. Positive interactions and information sharing should be encouraged between superiors and subordinates, and among various business departments, regarding the understanding of strategy, key business activities, resource requirements, and departmental value propositions throughout the innovation process. This can stimulate creative thinking among female STEM professionals and meet their resource needs for innovative work. Simultaneously, the accurate and transparent information obtained through informal control can encourage mutual learning and increase work engagement among female STEM professionals. Innovation activities should be business-oriented, without overemphasizing goal achievement, allowing flexibility for female STEM professionals’ innovative activities. Leaders should focus on risk factors and reasons for unmet innovation goals, promoting the enhancement of female STEM professionals’ innovation capabilities.

Secondly, managers should pay attention to the psychological impact of informal control on female STEM professionals. To maintain a positive work state for female STEM professionals, superiors should address role stress issues. Leaders should not only focus on female STEM professionals’ work willingness but also cultivate their professional and capability confidence. By strengthening cross-departmental communication, female STEM professionals should be guided to deeply understand and identify with business objectives, reducing conflicts in the decomposition of innovation goals. Superiors should communicate in-depth with subordinates to establish a transparent and reasonable performance indicator system that matches the job responsibilities of female STEM professionals, emphasizing both monetary rewards and spiritual recognition. Additionally, enterprises should utilize organizations like the Women Workers’ Committee to provide psychological support for female STEM professionals, increasing their sense of well-being and job satisfaction. Since COVID-19, the business environment has become more volatile, with the high-tech industry facing greater innovation demands and pressures (e.g., technological stress) than other sectors. High-tech enterprises should regularly and actively communicate with female STEM professionals to understand their resource needs and help them clarify their role positioning.

Thirdly, enterprises should leverage informal control to enhance female STEM professionals’ enthusiasm for participating in corporate management and decision-making. Managers should understand the resource needs of female managers, encourage them to express their ideas, support their in-depth learning and skill enhancement, and be adept at discovering and tapping into their innovation potential. Meanwhile, leaders should actively promote cooperation between male and female technology professionals, regularly hold meetings to exchange work experiences, summarize lessons learned, and facilitate multi-faceted cognition of work among employees, thereby elevating the overall innovation level of knowledge workers. Additionally, in recruitment and assessment processes, leaders should consider relaxing timeline requirements for female STEM professionals during their nursing periods and avoid situations where males dominate strategic choices and innovation decisions. Furthermore, enterprises can provide moderately flexible work hours and arrangements for female STEM professionals with children, based on their own goals, tasks, and actual work situations, enabling them to balance work tasks and family responsibilities. With the widespread application of internet technology, moderate work-from-home arrangements are increasingly being adopted by many organizations in practice.

### 5.4 Research limitations and prospects

This study exhibits several limitations. Firstly, the data collected is cross-sectional. Although the Harman single-factor test has been employed in this study to demonstrate that common method bias is unlikely to impact our research, establishing causality between variables remains challenging. Therefore, future research endeavors involve investigating causal relationships between variables using longitudinal data. Secondly, numerous factors influence the primary variables in this study, encompassing individual-level variables like manager age and work experience. However, the study only controlled for variables at the corporate level. Consequently, it would be advantageous to incorporate other potential influencing factors into the research framework in subsequent research. Furthermore, in deeper exploration, this study exclusively considered the impact of industry and childcare-related factors on informal control, role pressure, and innovation performance among female technology professionals in STEM fields. Future research initiatives could explore additional factors, such as the company life cycle (Krasyuk et al., 2019), to augment the scope of relevant studies.

## Data Availability

The raw data supporting the conclusions of this article will be made available by the authors, without undue reservation.

## Footnotes

^1^The term “STEM field female professionals” denotes women actively involved in occupations pertaining to science, technology, engineering, and mathematics (Glass et al., 2013).

^2^The Chinese Government attaches great importance to the Sustainable Development Goals, with particular attention to the message of gender equality (SDG 5). As can be seen from the relevant government website, the Chinese government has been making its own contribution to women’s equality (http://www.cnwomen.com.cn/, https://china.un.org/zh/take-action).

^3^The Credamo platform has been trusted by more than 2,000 universities and 3,000 companies worldwide, and has successfully completed more than 60,000 research projects.

^4^The division of high-tech and non-high-tech enterprises in the article is the industry defined when we send out questionnaires. The classification of high-tech and non-high-tech enterprises in this study is based on the standards outlined in the “Administrative Measures for the Recognition of High-tech Enterprises” jointly issued by the Ministry of Science and Technology, the Ministry of Finance, and the State Administration of Taxation of China in 2016.
