# Supplementary material for: The impact of informal control on the innovation performance of female technology professionals from the perspective of role pressure
Source: Front Psychol. 2024 Oct 29;15:1378056. doi: 10.3389/fpsyg.2024.1378056 (PMC11554465; doi:10.3389/fpsyg.2024.1378056)
Supplement: Supplementary file 1 [file Table_1.DOCX]

Supplementary Material

**Supplementary Table 1.** Informal control, role pressure, Innovation Performance, and Environmental turbulence scales.

|  | Question item | | Scale source |
| --- | --- | --- | --- |
| IC |  | To what extent do you agree with the following statements in the corporate work? | Jaworskiet al.（1993） |
|  | Social control | 1.Managers encourage employees to cooperate with each other |  |
|  |  | 2.corporate often carry out training and learning activities for their employees |  |
|  |  | 3.Your manager and you are constantly communicating in many forms |  |
|  |  | 4.Managers often gather the staff in your department together to thoroughly review and discuss all aspects of the work |  |
|  |  | 5.Managers encourage discussion and sharing of information about customer and industry trends |  |
|  | Cultural control | 1.Enterprise management work revolves around innovation, innovation is the core of management system |  |
|  |  | 2.The corporate work environment makes you feel like a part of it |  |
|  |  | 3.The employees of the enterprise have a high degree of participation in the work |  |
|  |  | 4.The company is committed to creating an employee culture of information sharing and mutual cooperation |  |
|  |  | 5.Management often communicates to employees that value creation is the core concept |  |
| IP |  | To what extent do you agree with the following statements in your work? | Murad and Fiona(2017) |
|  |  | 1.Create new ideas for improvements. |  |
|  |  | 2.Search out new working methods, techniques, or instruments. |  |
|  |  | 3.Acquire management approval for innovative ideas. |  |
|  |  | 4.Generate original solutions to problems. |  |
|  |  | 5.Transform innovative ideas into useful applications. |  |
| RP | role conflict | To what extent do you agree with the following statements in your work? | Rizzo et al.（1997）and Bedford et al（2022） |
|  |  | 1.I have to work on things that should be done differently. |  |
|  |  | 2.I receive incompatible requests from people. |  |
|  |  | 3. I do things that are accepted by one but not by another. |  |
|  |  | 4.I work on unnecessary things. |  |
|  | Role ambiguity | 1.I don't feel certain about how much authority I have |  |
|  |  | 2.I don’t know what my responsibilities are. |  |
|  |  | 3.I know that Idon’t have divided my time properly |  |
|  |  | 4.I don’t have clear, planned goals. |  |
|  |  | 5.I don’t know what is expected. |  |
| ET |  | Please indicate the extent to which your organization has experienced the following as a result of environmental disruption, such as COVID-19. | Becker et al. (2016);  Bedford et al. (2022) |
|  |  | 1.Have orders been affected? |  |
|  |  | 2.Have sales been affected? |  |
|  |  | 3.Has the ability of customers to pay been affected? |  |
|  |  | 4.Has the availability of capital been affected? |  |
|  |  | 5.Has reliability of supplies been affected? |  |
|  |  | 6.Has employee productivity been affected? |  |
